# Robotically Assisted Hysterectomy versus Vaginal Hysterectomy for Benign Disease: A Prospective Study

**DOI:** 10.1155/2013/429105

**Published:** 2013-07-07

**Authors:** M. Carbonnel, H. Abbou, H. T. N'Guyen, S. Roy, G. Hamdi, A. Jnifen, J. M. Ayoubi

**Affiliations:** Service de Gynécologie Obstetrique, Hôpital Foch, Faculté de Médecine Paris Ouest, 92150 Suresnes, France

## Abstract

*Objectives*. A prospective study was carried out to compare vaginal hysterectomy (VH) and robotically assisted hysterectomy (RH) for benign gynecological disease. *Materials and Methods*. All patients who underwent hysterectomy from March 2010 to March 2012 for a benign disease were included. Patients' demographics per and post surgery results were collected from medical files. A questionnaire was also conducted 2 months after surgery. *Results*. Sixty patients were included in the RH group and thirty four in the VH one. Operative time was significantly longer in the RH group (132.1 ± 5.7 versus 75.3 ± 6.7 min; *P* < 0.0001). Blood loss and length of hospital stay were significantly reduced: 47 ± 7 versus 125 ± 20 ml; *P* < 0.01, and 2.4 ± 0.1 versus 3.3 ± 0.2 days; *P* < 0.0001, respectively. Less pain was reported at D1 and D2 by RH patients, and levels of analgesia were lower compared to those observed in the VH group. No differences were found regarding the rate of conversion to laparotomy, intra- or postoperative complications. *Conclusion*. Robotically assisted hysterectomy appears to reduce blood loss, postoperative pain, and length of hospital stay, but it is associated with longer operative time and higher cost. Specific indications for RH remain to be defined.

## 1. Introduction

Many techniques of hysterectomy are being used in the surgical treatment of benign gynecological disease. Laparotomy is still the commonest and the easiest one, yet it is the most invasive [1]. Abdominal laparoscopic hysterectomy and vaginal hysterectomy (VH) should be preferred because they are minimally invasive. VH does not leave scars and it can be used in obese patients. However, it may be difficult in cases of enlarged uterus, nulliparous women, and in patients with pelvic adhesions; adnexectomy may also fail in case of upper abdominal adnexal masses [2]. Total laparoscopic hysterectomy reduces blood loss and postoperative pain; it is easer to make adnexectomy and adhesiolysis and is feasible in nulliparous women [3]. Laparoscopically assisted VH allows performing adnexectomy and adhesiolysis in hysterectomic procedures ended by vaginal approach which simplifies the laparoscopic time; however, such procedure is associated with greater postoperative discomfort compared to vaginal procedure [4, 5]. Robotically assisted surgery appears “pleasant” in this indication, overcoming the laparoscopic hysterectomy limits. The rotation of the instruments, the 3D visual approach, tremor reduction, operating comfort, and the intuitive pattern of the da Vinci robot significantly simplify surgical gestures. The da Vinci robot has been authorized for hysterectomy procedures in 2005 in the United States; hysterectomy is currently one of the most common surgical interventions worldwide [6]. To date, very few studies have compared the outcomes of robotically assisted hysterectomy (RH) and VH in benign gynecological disease. This paper presents our prospective comparison of these two approaches.

## 2. Methods

We carried out in our department of gynecology and obstetrics (Foch Hospital, Suresnes, France) a 2-year prospective study, from March 2010 to March 2012. All hysterectomies done for benign gynecological disease were included: 60 RH and 34 VH. Patients' demographics and medical characteristics were collected from the medical files: age, BMI, surgical indication, surgical history, menopausal status, and hormone replacement therapy were studied, as well as operative time, docking time, anesthesia, uterine weight, blood loss, transfusions, conversion to laparotomy, intra- and postoperative complications, and pre- and postoperative hemoglobin.

Two operators performed the RH and seven the VH. VH procedures were conventionally carried out with vicryl ligatures; RH procedures were performed using a uterine manipulator, and vaginal suturing was done using vicryl 1 continuous or interrupted sutures. A questionnaire was completed by all patients postoperatively, aimed to evaluate their pain at D0, D1, D2, and D3 using a visual analog rating 0–10 scale. The levels of analgesia, total morphine consumption, transit recovery delay, and the length of hospital stay (number of days) were also reported. Two months after surgery a questionnaire was completed by the patients regarding the duration of their work cessation, time to return to normal life, postoperative complications, pain, sexual life (unchanged, improved, or deteriorated compared with presurgery), and overall satisfaction regarding the intervention (dissatisfied, fairly satisfied, satisfied, and very satisfied). 

Quantitative variables were compared using nonparametric tests, and sample comparisons were performed by chisquare tests. The level of significance was *P* < 0.05. 

## 3. Results

Patients' characteristics are displayed in Table 1. In the VH group, indications were metrorrhagia associated with fibroma or endometrial hypertrophy in 26 cases, atypical hyperplasia in 4 cases, and high-grade dysplasia in 4 cases. Among RH patients there was a Benjamin syndrome in 20 cases, metrorrhagia induced by fibroma or endometrial hypertrophy in 27 cases, pain associated with adenomyosis in 3 cases, and atypical hyperplasia in 10 cases. To note that, patients were randomly classified into either group, except for those having Benjamin syndrome that were included in the RH arm.

Intraoperative data are displayed in Table 2. In the VH group, 2 patients underwent transfusion of 1 and 3 packed red blood cells (the 2 complications reported in this group) and 1 had laparoconversion induced by hemorrhage. In the RH group, the 2 reported complications were 1 bladder injury that occurred while detaching the vesicouterine cul-de-sac (patient with a history of 3 cesarean sections) and 1 injury of the small intestinal serosa that occurred during open laparoscopy, sutured by one vicryl 2.0. Five adhesiolyses were carried out in this group.

Table 3 presents the postoperative results until hospital discharge. At D3, 7 patients had left hospital in the VH group (20%) and 23 in the RH group (40%). No postoperative complications were reported in the VH group while 1 occurred in one RH patient: an abscess of Douglas pouch occurring 10 days after surgery and necessitating antibiotherapy along with a 5-day hospital stay without surgical reintervention. 

The results obtained by the questionnaire completed 2 months after surgery are displayed in Table 4. In the VH group 28 questionnaires (82%) have been completed and 41 (70%) in the RH one. No difference was observed between the two groups regarding sexual life. In the VH group, among the 16 patients reporting a sexual activity before and after surgery, 8 evaluated it as unchanged, 4 worsened, and 4 improved. In the RH group, among the 20 patients reporting a sexual activity before and after surgery, 16 evaluated it as unchanged, 1 worsened, and 3 improved. 

## 4. Discussion

Robotically assisted surgery offers advantages over laparoscopy in hysterectomy procedures for benign disease. The princeps series of Payne and Dauterive [7] showed beneficial results regarding uterine weight, operative time in the 25 last procedures (series of 100 cases), blood loss, laparoconversions, and hospital stay duration. This author confirmed such results in a meta-analysis [8]. The rate of vaginal cuff dehiscence has been probably overestimated in the first series [9]; it appears to be 1.5% like that observed with laparoscopy [10]. We had no cases of dehiscence in our series: only one case of pelvic abscess that resolved after antibiotherapy. 

A comparative study of RH and laparoscopic-assisted VH showed that the robotic procedure reduces the operative time and duration of hospital stay with less blood loss [11]. Very few studies have compared RH and VH [12–15]. Matthews et al. carried out a retrospective analysis of the various surgical approaches used in their department during the first year after robotic equipment was introduced in this unit [12]. They observed beneficial results associated with the robot regarding blood loss, transfusion rate, and infection rate. In another retrospective series, Landeen et al. [15] compared all surgical approaches for hysterectomy; they underline less blood loss with the robot and reduced hospital stay, while VH was associated with a shortened operative time and reduced cost of the procedure. The two other comparisons were reported in congress abstracts [13, 14]. 

We found no randomized study or prospective study on this comparison. Our results are in accordance with those reported in the literature regarding blood loss and duration of hospital stay. We observed also a lengthened operative time with the robotic procedure. Our study reports no significant difference between the two procedures regarding intra- and postoperative complications; in fact, hysterectomy in benign disease is usually associated with a low incidence of complications, and no difference could be evidenced with such small sample. 

We also focused on postoperative pain, a fact poorly present in the literature, although some authors have underlined the beneficial outcome of laparoscopy over VH regarding postoperative pain [4]. Our study reports less postoperative pain associated with the robotic approach at D1 and D2 on the rating scale and lower analgesic level at D2. Such results were not seen at D3, probably biased by the discharge of a great number of RH patients at D2. We observed no difference in terms of morphine consumption. Morphine-like agents are primarily used in the recovery room and may have been overused at first interventions carried out in the department, making our results possibly biased. The questionnaire completed 2 months after surgery shows no significant differences between groups and reveals a significant number of lost-to-follow-up patients. The main bias of our series is the lack of randomization of all patients. In fact, populations were different with younger patients, lower parity data, and more frequent nonconservative hysterectomies in the RH group. This bias was due to surgical indications. Benjamin syndromes were young and had smaller uterus. But they were nullipara, and it was very important for them to undergo ovariectomy. So hysterectomy was robotically assisted for this indication in all cases (1/3 of indications of RH group) in order to avoid laparotomy. Therefore we have to continue evaluation in the future with information collected prospectively and probably with randomized methodology We have not studied the related costs, although this represents a major disadvantage of the robotic surgery. The costs related to robotic surgery are higher than those related to the laparoscopic and vaginal approaches [16] but lower than laparotomy-related operative cost. 

The advantages presented by the robotic surgery over the vaginal approach in hysterectomy are counterbalanced by its higher operative cost and lengthened operative time. To date, it does not seem reasonable to systematically use robotics in all hysterectomies, but the robotic procedure presents significant interest in that it allows preventing laparotomy and laparoscopic-assisted VH. Such technique could be considered in complex diseases (enlarged uterine volumes, obese patients, etc.) [17] until the reduction of its cost which should help its diffusion.

## Figures and Tables

**Table 1:** Demographics and characteristics of the population expressed as mean ± SD and number (%).

	RH (*n* = 60)	VH (*n* = 34)	*P *
Age (years)	43.7 ± 1.7	51.2 ± 1.6	0.06
BMI (kg/m²)	24.8 ± 0.5	26.5 ± 1.3	NS
Gestity	1.7 ± 0.3	2.7 ± 0.3	0.02
Parity	1.3 ± 0.2	2.3 ± 0.2	0.01
Menopause	14 (23%)	9 (26%)	NS
HRT	7/14 (50%)	2/9 (22%)	NS
History of laparotomy	17 (28%)	7 (20%)	NS
Nonconservative hysterectomy	43 (72%)	19 (29%)	0.02
Interadnexal hysterectomy	17 (28%)	24 (71%)	0.01

SD: standard deviation, NS: nonsignificant, HRT: hormone replacement therapy.

**Table 2:** Interoperative results expressed as mean ± SD and number (%).

	RH (*n* = 60)	VH (*n* = 34)	*P *
pAnesthesia time (min)	195.8 ± 6.4	115.3 ± 7	<0.0001
Operative time (min)	132.1 ± 5.7	75.3 ± 6.7	<0.0001
Trocar placement time (min)	9.5 ± 0.5		
Docking time (min)	7 ± 0.6		
Console time (min)	94 ± 6		
Uterine weight (g)	136 ± 14	226.7 ± 31.6	0.004
Blood loss (mL)	47 ± 7	125 ± 20	<0.01
Transfusions	0	2 (6%)	NS
Laparoconversion	0	1	NS
Complications	2 (3%)	2 (8%)	NS

SD: standard deviation, min: minutes, mL: milliliters, g: grams, NS: nonsignificant.

**Table 3:** Postoperative results expressed as mean ± SD and number (%).

	RH (*n* = 60)	VH (*n* = 34)	*P *
Pain on the VAS, D0	4.7 ± 0.4	5.1 ± 0.4	NS
Analgesic level, D0	2.5 ± 0.1	2.3 ± 0.1	NS
Pain on the VAS, D1	3.1 ± 0.3	4 ± 0.3	0.03
Analgesic level, D1	1.8 ± 0.4	1.8 ± 0.1	NS
Pain on the VAS, D2	2.2 ± 0.3	3.2 ± 0.3	0.04
Analgesic level, D2	1.3 ± 0.1	1.7 ± 0.1	0.04
Pain on the VAS, D3	1.8 ± 0.3	2.3 ± 0.2	NS
Analgesic level, D3	1.2 ± 0.2	1.4 ± 0.1	NS
Time to transit return (days)	1.3 ± 0.1	1.5 ± 0.1	NS
Total morphine consumption (mg)	7.9 ± 1.4	5.6 ± 1.2	NS
Difference in hemoglobin level (g/dL)	1.2 ± 0.1	0.9 ± 0.1	NS
Duration of hospital stay (days)	2.4 ± 0.1	3.3 ± 0.2	<0.0001

VAS: visual analog scale, SD: standard deviation, dl: deciliters, g: grams, NS: nonsignificant.

**Table 4:** Results of the questionnaire completed 2 months after surgery expressed as mean ± SD and number (%).

	RH (*n* = 60)	VH (*n* = 34)	*P *
Duration of work cessation (days)	30.9 ± 2.5	35.9 ± 3.5	NS
Return to normal life (days)	20.7 ± 2.9	17.9 ± 3.3	NS
Dyspareunia	1/20 (5%)	2/17 (12%)	NS
Pelvic pain	12 (29%)	9 (28%)	NS
Analgesic level	0.6 ± 0.1	0.5 ± 0.1	NS
Very satisfied	31 (75%)	22 (70%)	NS
Satisfied	6 (15%)	6 (18%)	NS
Fairly satisfied	2 (5%)	2 (6%)	NS
Dissatisfied	2 (5%)	2 (6%)	NS

NS: nonsignificant.
